# Supplementary material for: Seeking ambulance treatment for ‘primary care’ problems: a qualitative systematic review of patient, carer and professional perspectives
Source: BMJ Open. 2017 Aug 3;7(8):e016832. doi: 10.1136/bmjopen-2017-016832 (PMC5623409; doi:10.1136/bmjopen-2017-016832)
Supplement: Supplementary file 1 [file bmjopen-2017-016832supp001.pdf]

Appendix – Search String Example, PubMed

byDateRange: 01/01/1980-30/06/2014;
(((((((((((((((((emergency medical services
[mesh: EXP Emergency Health Services; Emergency Services, Medical; Emergicentres; Medical Services, Emergency; Prehospital Emergency Care; Services, Emergency Medical])
OR emergency care* [tiab])
OR prehospital* [tiab])
OR pre#hospital* [tiab])
OR pre*hospital* [tiab])
OR EMS [tiab]) OR emerg* serv* [tiab])
OR ambulances
[mesh: EXP Emergency Mobile Units; Mobile Emergency Units])
OR ambulance* [tiab])
OR 999 [tiab])
OR *111 [tiab])
OR 911 [tiab])
OR paramedic* [tiab])
OR emerg* med* tech* [tiab])
OR EMT [tiab])
OR emergency treatment
[mesh: EXP Emergency Therapy; Therapy, Emergency]) OR emergency* [tiab])
OR emergencies [mesh])
AND
((((((((primary health care
[mesh: EXP Primary Care; Primary Healthcare])
OR primary care* [tiab])
OR primary *care [tiab])
OR family medicine* [tiab])
OR family practice* [tiab])
OR after hours care
[mesh: EXP After Hours Care; Out Of Hours Medical Care])
OR OOH [tiab])
OR out of hours* [tiab])
OR out#of#hours [tiab])
OR primary care sensitive [tiab])

OR primary care problem [tiab])
OR general practice
[mesh: EXP General Practitioners]))
OR (((((((social care* [tiab])
OR non*serious* [tiab])
OR non*emergenc* [tiab])
OR non*urgent [tiab])
OR minor* [tiab])
OR routine* [tiab])
OR avoid* [tiab])
OR inappropriate* [tiab])
OR unnecessary* [tiab])
{1381}
