# Supplementary material for: Seeking ambulance treatment for ‘primary care’ problems: a qualitative systematic review of patient, carer and professional perspectives
Source: BMJ Open. 2017 Aug 3;7(8):e016832. doi: 10.1136/bmjopen-2017-016832 (PMC5623409; doi:10.1136/bmjopen-2017-016832)
Supplement: Author's manuscript [file bmjopen-2017-016832.draft_revisions.pdf]

BMJ Open

Seeking ambulance treatment for ‘primary care’ problems: a qualitative synthesis of patient, carer and professional perspectives.

Journal:	*BMJ Open*
Manuscript ID	bmjopen-2017-016832
Article Type:	Research
Date Submitted by the Author:	14-Mar-2017
Complete List of Authors:	Booker, Matthew; University of Bristol, School of Social and Community Medicine<br>Purdy, Sarah; University of Bristol, Centre for Academic Primary Care<br>Shaw, Ali; University of Bristol, School of Social and Community Medicine
**Primary Subject Heading**:	Emergency medicine
Secondary Subject Heading:	General practice / Family practice, Health services research, Qualitative research
Keywords:	Ambulance, PRIMARY CARE, Urgent Care, Decision-making

SCHOLARONE™
Manuscripts

For peer review only

Seeking ambulance treatment for 'primary care' problems: a qualitative synthesis of patient, carer and professional perspectives.

Matthew J Booker [a]

Sarah Purdy [a]

Alison R G Shaw [a]

Author Affiliations: [a] Centre for Academic Primary Care, School of Social & Community Medicine, University of Bristol, Bristol, UK

Corresponding author at:

Matthew J Booker, Office 1.07, School of Social and Community Medicine, University of Bristol, Canynge Hall, Whatley Road, Bristol, UK BS8 2PS

Matthew.Booker@Bristol.ac.uk

+44 (0)117 9287305

Word Count: 4073

For peer review only

For peer review only - http://bmjopen.bmj.com/site/about/guidelines.xhtml

ABSTRACT

Objectives: Understanding patients' decision-making processes is key to supporting appropriate access to urgent-care. This paper provides a detailed evidence synthesis of the experience of seeking and receiving ambulance treatment for problems potentially treatable in a 'primary care' setting.

Design: A systematic search identified qualitative studies that explored patient, carer and professional perspectives around the use of ambulances for 'primary care' problems. An analytical thematic synthesis was undertaken.

Results: Patients define situations worthy of 'emergency' ambulance use according to complex socio-emotional factors, as well as experienced symptoms. Deciding to call an ambulance is a process shaped by practical considerations and an (often) strong emotional component, which is can be influenced by the views of caregivers. Clinicians and patients conceptualise 'urgency' in different ways. There is a strong negative emotional response when callers perceive they are being labelled as 'inappropriate' users of ambulances, which may have implications for their future relationship with urgent-care services.

Conclusions: When responding to a request for 'emergency' help for a low-acuity condition, urgent-care services need to be sensitive to how the patient's emotional and practical perception of the situation may have shaped their decision-making. Labelling those who use ambulance services for primary care problems as 'inappropriate' may be unhelpful.

Keywords

Ambulance; Urgent Care; Primary Care; Decision-making;

For peer review only - http://bmjopen.bmj.com/site/about/guidelines.xhtml

STRENGTHS AND LIMITATIONS OF THIS STUDY

- This evidence synthesis expressly focuses on the experiences of providing and receiving ambulance-care for ‘primary care sensitive’ clinical problems, which are forming an increasing part of pre-hospital care workload.
- The methods of qualitative synthesis build on previous literature mapping work, to offer a more nuanced appreciation of the complexities experienced by patients, carers and professionals in seeking ambulance treatment.
- The majority of qualitative evidence is drawn from ambulance systems in more economically developed countries with an established primary care model, and in adult populations.

For peer review only

For peer review only - http://bmjopen.bmj.com/site/about/guidelines.xhtml

INTRODUCTION

Despite origins as services for those with acute medical emergencies and injuries,[1] the majority of contacts with ambulance organisations are no longer for serious immediately life-threatening conditions.[2] Calls to the emergency ambulance service in the UK have been rising over recent years at 7% per annum,[3] and are increasingly for conditions that could potentially be managed by a primary care provider.[4] The proportion of ambulance calls for mental health conditions and social situations are also rising for reasons that are poorly understood.[4]

A recent systematic mapping review by the authors identified a number of factors that may be associated with ambulance use for problems that could be managed in primary care, including markers of deprivation, minority status and certain social circumstances.[5] Much of the previous literature exploring help-seeking for such problems in the context of ambulance use has focussed on the so-termed ‘inappropriate’ use of the service, usually defined from the health care professional perspective. However, defining use in this way neglects to appreciate the complex processes that are likely to underlie help-seeking behaviours and attitudes in these user groups. Indeed, healthcare professionals themselves lack consensus as to what constitutes ‘inappropriate’ use of emergency healthcare.[6] There is increasing acceptance that labelling such ambulance services use as inappropriate fails to recognise the context of the request for assistance, and is unhelpful for developing practical solutions. Additionally, making the decision to call an ambulance is not always the ‘easy’ option for many service users, as previous qualitative research by the authors [7] suggests that many emergency calls are preceded by a substantial degree of internal conflict on the part of the caller about the best course of action.

For peer review only - http://bmjopen.bmj.com/site/about/guidelines.xhtml

An established body of sociological research has sought to unpick what underpins illness behaviour and help-seeking in general. The more established models of illness and help-seeking acknowledge a complex interplay between biological predisposition to illness, experienced symptomatology, learned behaviour patterns, attributional predispositions, situational influences, and the organisational incentives and secondary benefits of the health care system itself.[8] Recent work examines how help-seeking and decision-making models may apply to some urgent care settings, such a GP Out-of-Hours [9] and Emergency Departments,[10] but there has been a lack of attention to how patients and carers conceptualise the need for ambulance care specifically. There is very limited understanding of how help-seeking models apply to situations where the request for ambulance care is for a problem that is potentially manageable in a primary care setting.

This paper, therefore, seeks to provide a more detailed and nuanced account of the experience of seeking and receiving ambulance treatment for ‘primary care sensitive’ conditions, by synthesising relevant data from qualitative studies of patients, carers and professionals.

For peer review only

For peer review only - http://bmjopen.bmj.com/site/about/guidelines.xhtml

METHODS

An electronic systematic literature search was undertaken on the following databases: MEDLINE, EMBASE, PsychInfo, CINAHL, Health Management Information Consortium and Health Management Information Service. Google Scholar and Web of Science searches were undertaken to identify reports not captured by the above. Additional searches of OpenSigle, EThOS and DART databases were undertaken. The comprehensive systematic strategy was complemented by hand searches of key journals. This literature search formed the basis of a systematic literature mapping exercise,[5] the protocol of which has been published on the PROSPERO register (reference CRD42014009108). Search terms were developed iteratively by consensus discussion amongst the research team and a medical subject librarian. Initial searches were run for papers published between January 1980 and June 2014, with a further update search covering the period June 2014 to January 2016.

Inclusion and Exclusion Criteria

The inclusion criteria for the systematic mapping review [5] were articles published in the English language, reporting the findings of research exploring patient, carer, healthcare professional or health service management perspectives and experiences of interaction with ambulance services for 'primary care sensitive' problems. The definition of a 'primary care sensitive' condition could be from any perspective, assigned prospectively or retrospectively. A 'primary care sensitive' contact could include explicit reference to terms related to primary care or family medicine, or could be defined by reference to a comprehensive list of indicator conditions developed in conjunction with a medical subject librarian. Studies that reported on any stage of the

For peer review only - http://bmjopen.bmj.com/site/about/guidelines.xhtml

interaction (from telephone call to treatment or transport) were of interest. Studies reporting on routine primary care only were excluded.

For this qualitative synthesis, further inclusion criteria were applied to identify papers reporting qualitative research. Papers were included if the data collection and analysis were wholly qualitative. Mixed-methods papers were included if they reported identifiable components that had used qualitative data collection and analysis methods. Papers were screened by two researchers (MB, AS) independently, with all excluded papers further reviewed by all researchers to ensure agreement.

Extracting, Coding and Analysing the Data

A thematic synthesis was undertaken, following the approach described by Thomas and Harden.[11] All verbatim text in the 'results' and 'discussion' sections (or equivalent) of papers was treated as data for the purposes of this analysis. Data were loaded electronically into NVivo software (version 11), and a process of line-by-line open coding undertaken. Results, which often included quotations or descriptions of themes, were coded alongside authors' interpretive statements in the discussion section. The synthesis took the form of a three-level inductive thematic analysis: open line-by-line coding of the primary studies, organisation into groups of descriptive themes, and finally the development of overarching analytical themes.[12] The ENTREQ statement was followed throughout the data collection, appraisal and synthesis.[13]

There are a range of methodological approaches to handling and analysing data as part of a qualitative synthesis,[13] including meta-theoretical and meta-ethnographic approaches that draw upon grounded theory and line-of-argument principles to

For peer review only

synthesise ‘key concepts’ (e.g.[14]) and critical interpretive methods resulting in synthetic constructs. (e.g.[15]) The line-by-line thematic synthesis approach [11] was used in this analysis. With philosophical foundations in critical realism, this approach permits a holistic understanding of the described phenomena through a more flexible way that ‘free’ first levels codes can be applied to various types of source data in the included studies. Table 1 provides example extracts from the analysis to illustrate this process of coding ‘up’ from free codes, to descriptive then analytical themes. MB led on this process with regular research meetings between all authors to verify coding and agree themes by consensus.

Original Text	Free Codes	Descriptive Themes	Analytical Themes
That’s the worst of staying out here in the wilds out here because they speak about this nine minutes and stuff o’ this kind, but that is impossible staying oot here, like	3.45 Difficult/impossible for ambulances to arrive quickly/meet response time targets in geographically remote areas.<br>3.46 Acknowledgement that living in rural area necessarily has (disadvantage) of time delay in getting emergency care.	RURALITY AND REMOTENESS: There are consequences of living in a rural or remote area in terms of how quickly ambulance care can be accessed. Patients recognise and even accept this constraint, and adapt their expectations and the way they decide to physically access care, and the way in which they get to the location of treatment.	**Practical Domain** – whatever the perceived health need may be, and regardless of who may be involved assessing it, there are physical practicalities that sometimes override all other aspects of the decision-making process and take precedence. These can be related to geography and space, access to modes of transport, physical limitations of an individual’s capability to care for themselves or the perceived need for immediate, expert care that can only be provided by ambulance staff.
If it was very severe, I would get my husband… to drive me to the hospital	3.72 Very severe illness requires hospital treatment.<br>3.73 Assistance from relative required to access appropriate care.<br>3.74 Decision to drive/make own way to hospital instead of call and wait for ambulance.		
I could have gone in my car… I would have made a lot of work for my relatives, which I don’t think is right.	2.92 Health condition such that could have gone by car, but active decision not to due to convenience.<br>2.93 Assistance from relative required to access care<br>2.94 Reluctance to inconvenience relatives as a result of own illness.	TRANSPORT: role of seeing the ambulance service as a transport when other options are discounted as unsuitable or inconvenient, even when they may be entirely suitable as the clinical condition is relatively minor.	**Process Domain**: **being influenced by others** – perception of whether one would be a burden on one’s relatives shapes the decision making process about how to access urgent treatment (subtle or ‘invisible’ influence of relatives).

For peer review only - http://bmjopen.bmj.com/site/about/guidelines.xhtml

Table 1: Example data extracts demonstrating the hierarchical coding process

Assessment of Quality

The assessment of quality in qualitative syntheses is much debated and there is little consensus about whether to do quality appraisal and how to do it.[11] Some qualitative methodologists argue that quality should not or cannot be meaningfully measured at all,[16,17] particularly if the synthesis aims to include all relevant existing data, however collected or reported. The authors of this synthesis take the view that while relevance of a paper to the review may outweigh reporting quality, it is helpful to attempt to assess quality in a consistent and transparent manner, to allow the reader to make judgements about how particular papers have contributed to a synthesis. In this synthesis, whilst studies were not excluded on the basis of a quality assessment alone, recognition of the quality limitations of included papers enabled a sensitivity analysis to be performed as described below.

A number of tools have been developed to assist in the systematic appraisal of quality in qualitative research.[18] Many of these are limited to informing the inclusion/exclusion process, and some have been criticised for blending different aspects of quality assessment under too few headings. The Critical Appraisal Skills Programme (CASP) checklist [19] is often utilised for quality assessment in qualitative syntheses, prompting assessment of a paper against a number of items related to the purpose, design, conduct and reporting of qualitative research. A modified CASP checklist was used in this synthesis to assess included papers under a number of headings: overall appropriateness of the qualitative methodology, credibility, transferability, dependability, and confirmability, including detail of the reporting. Whilst there is considerable debate about assigning numerical ‘scores’ to such quality appraisals, the synthesis team adopted the four-tiered quality summary

For peer review only - http://bmjopen.bmj.com/site/about/guidelines.xhtml

score described by Downe [20] to report the output of this quality assessment in a transparent manner. The final quality score, described in Table 2, was assigned by consensus amongst the research team following the appraisal process described above.

Score	***Description***
1	No or few flaws: credibility, transferability, dependability and confirmability is high.
2	Some flaws: unlikely to affect the credibility, transferability, dependability or confirmability.
3	Some flaws, which may affect the credibility, transferability, dependability, and/or confirmability
4	Significant flaws, which are very likely to affect the credibility, transferability, dependability and/or confirmability

Table 2: Quality summary scores for qualitative studies (adapted from Downe [20])

To enable judgement about how individual papers of varying reporting quality had contributed to the synthesis, a form of sensitivity analysis was undertaken as described by Thomas and Harden,[11] whereby the themes arising from each of the included papers were supressed from the analysis. This was achieved by removing all coded data from the NVivo dataset, paper by paper, and comparing the model against the full analysis. There was no substantial impact on the top-level conceptual model as a result of this, indicating that the final model was not disproportionately shaped by studies of any particular quality.

For peer review only - http://bmjopen.bmj.com/site/about/guidelines.xhtml

The Quality-Relevance (Q-R) Plot

Assessing relevance of qualitative studies in a synthesis is also an area of some debate, and often includes assessment of some parameters that could arguably overlap with measurements of quality.[16] For the purposes of this qualitative synthesis, the concept of relevance is defined as applicability of the study's findings to the authors' health system and infrastructure, namely a (broadly) nationally homogenous ambulance service operating within a health infrastructure with an established primary-care model. As such, relevance was assessed – again by consensus – into one of three categories, as summarised in Table 3:

A	Concerning UK Ambulance Services and/or UK 'Primary Care' Model
B	Concerning Westernised (but non-UK) Ambulance Service and/or non-UK 'Primary Care' based model of healthcare
C	Concerning non-Westernised Ambulance Service and/or non-Primary Care based model of healthcare.

Table 3: Categorisation of study relevance

In order to visualise the spread of included studies by both quality and relevance, the authors developed the concept of the Quality-Relevance (Q-R) plot (Figure 1).

Figure 1: 'Quality-relevance plot' of included studies

For peer review only - http://bmjopen.bmj.com/site/about/guidelines.xhtml

RESULTS

Search results

The literature search identified 1458 references (n=1424 during the initial search, and n=34 in the update search). After duplicate suppression and removal of irrelevant and incorrectly cited documents, first level screening against the inclusion/exclusion criteria resulted in 33 studies remaining (n=31 from the initial search and n=2 from the update search). Second level screening for qualitative methods content resulted in 6 papers (n=5 from the initial and n=1 from the update searches) meeting full inclusion criteria for the qualitative synthesis. The characteristics of these studies are summarised in Table 4. Figure 1 summarises the authors' assessment of quality and relevance as described above.

Paper	Year	Setting	Sample	Study Methodology
Booker et al. [7]	2013	UK Ambulance Service	16 adult participants (patients and carers)	Qualitative semi-structured interview study; thematic analysis
Ahl C et al. [21]	2006	Swedish Ambulance Service	20 adult participants (patients)	Qualitative interview study, content analysis.
Campbell et al. [22]	2006	Scottish Primary Care	78 adult participants (patients)	Qualitative semi-structured interviews and focus groups, inductive thematic analysis
Rantala et al.	2015	Swedish Ambulance	12 adult participants	Qualitative open-ended interviews, inductive

For peer review only - http://bmjopen.bmj.com/site/about/guidelines.xhtml

[23]		Service	(patients)	phenomenological hermeneutic analysis
Porter et al. [24]	2007	UK Ambulance Service	25 adult participants (paramedics)	Qualitative focus group study; thematic analysis
Togher et al. [25]	2014	UK Ambulance Service	30 (22 patients, 8 spouses)	Qualitative interview study, thematic analysis and mapping.

Table 4: Characteristics of the studies included in the thematic synthesis

From Open Codes to Descriptive Themes

Following the process of free coding, a total of 23 descriptive themes were developed iteratively by repeated cycles of reductive grouping of codes until no additional discrete categories were required to fully describe the free-code dataset. As both verbatim primary data transcriptions and authors' interpretations were included as 'data' in this exercise, these descriptive themes already have a degree of interpretation included in them. These descriptive themes were then organised into nine, related descriptive theme groups. The relationship is shown in Table 5.

For peer review only

For peer review only - http://bmjopen.bmj.com/site/about/guidelines.xhtml

Receiving treatment for a complaint that the caller knows is minor or non-serious.	Needing and receiving **treatment** urgently
Receiving immediate, life saving treatment for a condition the caller suspects is serious or life-threatening.	
Skillset and capabilities of the ambulance staff providing treatment	
Using the ambulance as a means of quick transport to hospital (when there is a medical need)	The **transport** role of the ambulance service
Using the ambulance as a 'convenient' mechanism of transport to hospital (with or without medical need)	
The impact of rurality and geographic remoteness on the care available when needed	**Rurality and remoteness** and the impact on receiving ambulance care
The impact or rurality and geographic remoteness on personal expectations and choices about accessing care	
Patient uncertainty about the seriousness of their health conditions, and whether there is urgent medical need	**Uncertainty** about own health and the choice for ambulance care
Patient uncertainty about how to access the most appropriate health care service	
Patient uncertainty about the impact of their actions on others	
Uncertainty about making a decision on behalf of someone else (relative or friend)	
Need for personal reassurance that there is no underlying serious health problems	**Reassurance** about the absence of serious health problems
Need for reassurance that there is a legitimate need to access ambulance care	
Providing information to medical professionals to allow them to make appropriate decisions	Taking or handing over **control** of the situation
Taking decisive action to manage an intolerable circumstance	
Compassion provided by ambulance staff (in comparison to other healthcare groups)	Experience of **compassion** during contact with the ambulance service
Compassion of friends and relatives in times of illness when calling an ambulance	
Influence of others (friends and relatives) when asked for their opinion	Being **influenced** by the opinions and experience of others about ambulance care
Influence of others (friends and relatives) through their perceived opinions, even though the person may not present or directly consulted	
The need to take responsibility for one's own healthcare	Taking **responsibility** and being **empowered** to manage one's urgent health needs
The need to take responsibility for the healthcare of another (friend or relative)	
Empowerment to manage own healthcare	
Empowerment to access ambulance care appropriately	

Table 5: Relationship between descriptive themes and thematic groups

For peer review only - http://bmjopen.bmj.com/site/about/guidelines.xhtml

Development of Analytical Themes

By analysing patterns in the free codes and descriptive themes within and across t the nine thematic groups, a number of cross-relationships between groups were identified. Through a process of comparing the theme groups and their constituent descriptive themes, three overarching analytical themes were identified. These analytical themes conceptualise ‘domains’ of experience that are shared by all involved in the event of ambulance treatment for primary care sensitive conditions: practical domain, emotional domain and process domain.

The **practical** domain contains descriptive themes relating to the practicalities of needing and receiving treatment, including themes associated with access issues and transport. The **emotional** domain brings together descriptive themes concerning the emotions involved in needing, calling for, receiving and providing ambulance treatment. The **process** domain is a conceptually distinct and central domain, referring to the processes by which patients, carers and professionals try to integrate the practical needs and limitations of the situation with the emotional challenges associated with illness and help-seeking. Figure 2 summarises how the three analytical themes are related.

Figure 2: Relationships between analytical themes

For peer review only - http://bmjopen.bmj.com/site/about/guidelines.xhtml

The practical domain

This analytical theme unites descriptive themes relating to the practicalities of asking for or providing ambulance care. Whilst the practical aspects (including logistic difficulties and convenience) may not always be the first consideration, many of the themes include some aspect of patients and carers 'weighing up' how practical the use of the ambulance service (or alternatives) are for their perceived needs.

In many instances, patients appear to know, broadly, what is wrong with them and what treatment is required, but do not appreciate that the problem is out-of-scope of the ambulance service. Sometimes patients appear to genuinely expect the ambulance service to treat minor ailments and injuries in preference to GP services – a belief that appears rooted in misunderstanding of how urgent care services are structured. In these circumstances, the call to the ambulance service is made in 'good faith' that this is the right way to access treatment. Sometimes, however, patients acknowledge that the condition is not life threatening, but feel that there are genuine practical issues that would prevent appropriate treatment elsewhere, in what they see as a logical and reasoned attempt to arrive at the most sensible outcome:

"The doctor would find it very difficult to do anything useful, I think, just with me describing the symptoms of a water infection over the telephone". [Booker et al., participant quotation]

"The doctor on the phone said [it may be] early appendicitis. You are bound to need blood tests and scans and stuff… so I just called the ambulance to get straight up to hospital." [Booker et al., participant quotation]

The logic behind these decisions is not fundamentally unsound, but there is a lack of understanding of the implications of these access choices on the wider system.

For peer review only - http://bmjopen.bmj.com/site/about/guidelines.xhtml

Practical issues appear to be viewed in differing ways, with often minor logistic barriers being used as a justification for using ambulance services. The theme of the ambulance service fulfilling a transport role is one such example, with the idea of getting to hospital more quickly or more easily being central. There are examples of when patients articulate that ambulance transport is more convenient, if not strictly a necessity:

"I could have gone in my car… I would have made a lot of work for my relatives, which I don't think is right". [Ahl et al., participant quotation]

Thus, while not always overt in the primary data, there were connections between data within the descriptive theme about 'convenience' of ambulance service use and data within the descriptive theme relating to perceived impact upon others around the patient. Thus, 'convenience' extends to include patients not wishing to be a burden on their carers and relatives.

Convenience can also be viewed from the perspective of physical and geographic isolation. Being geographically remote was something that some patients acknowledged as impacting upon what kind of service they might expect from the ambulance:

"That's the worst of staying out here in the wilds out here because they speak about this nine minutes and stuff o' this kind, but that is impossible staying oot here, like." [Campbell et al., participant quotation]

This differing geography resulted in those living in urban areas taking a notably different course of action to those living in rural areas, seemingly as a result of the

For peer review only - http://bmjopen.bmj.com/site/about/guidelines.xhtml

overt realisation of the impact of the 'remoteness' on the likelihood of getting a timely ambulance response:

"If it was very severe, I would get my husband… to drive me to the hospital". [Ahl et al., participant quotation]

"Patients in rural areas consult primary health care less frequently than urban patients… and are more likely to call their own general practitioner in emergencies" [Ahl et al., author interpretation]

Themes in the practical domain also allude to an undertone of frustration that patients can experience if they do not initially receive the care that they expect. Being unable to 'get through' to the right person first time seems to link with the negative emotional responses associated with inability to get reassurance, worsening uncertainty and anxiety and a feeling that the 'system doesn't care'. Free codes relating to practical barriers and access issues frequently co-existed with codes describing negative emotions:

"The walk in centre just sends you up to hospital if they can't do anything. So you end up, like, waiting 3 hours twice!" [Booker et al., participant quotation]

"The district nurse said she couldn't help. The doctor's surgery couldn't help. The hospital clinic couldn't help. After that many calls, you just snap?" [Booker et al, participant quotation].

Within the emotional domain there were also descriptive themes and codes relating to the *potential* treatment that the ambulance service could provide, if necessary. Strong links existed between patients' knowledge of practical treatment that crews could provide, and the emotional themes of reassurance and uncertainty. This suggests that it is as much the *potential* and *perceived* capacity of the ambulance

For peer review only - http://bmjopen.bmj.com/site/about/guidelines.xhtml

service to deliver life-saving treatment that is valued, and alludes to the idea of risk-avoidance or risk-minimisation when selecting which urgent care service to access.

The Emotional Domain

This domain encompassed descriptive themes relating to how people responded emotionally to the experience of needing or receiving ambulance treatment. Descriptive themes in this domain were organised into three groups: the issue of uncertainty about their condition or health, the need for (and impact of) reassurance, and the effect of compassion shown by others during the ambulance service contact. Analysis of emotion-related themes across the studies supports the ideas that the emotional response was not always congruent with actual medical need.

The descriptive theme of uncertainly was often associated with codes referencing anxiety, but appears to more broadly reflect the idea of dealing with the unknown:

"I just needed someone there quickly… to tell me whether it was something serious?". [Booker et al., participant quotation]

"A sense of insecurity and vulnerability emerged due to now knowing what was wrong" [Rantala et al., author interpretation]

Within the descriptive theme of uncertainty there existed a continuum of emotions, ranging from codes associated with minor indecision through to despair. There was no clear or consistent link with the actual type or severity of clinical condition experienced by these patients, suggesting that extreme emotional responses were apparent in even relatively minor medical problems. Importantly, uncertainty appears

For peer review only - http://bmjopen.bmj.com/site/about/guidelines.xhtml

to be experienced by ambulance crews as well, particularly around how best to manage non-urgent presentations:

"Even where the crew did not think that the patient should go to hospital and the patient did not want to go, crew members were still bothered by the fear of a possible comeback if it turned out to be the wrong decision". [Porter et al., author interpretation]

This is particularly noteworthy, as the descriptive theme of reassurance links equally with themes that rest principally in the practical domain and themes that appear purely emotion-driven. This suggests the potential powerful effect of reassurance – perhaps almost therapeutic - on helping patients gain practical *and* emotional control of the situation they are experiencing:

"I felt so ill that I thought I was about to become unconscious, but knowing that someone is on the way meant I was not worried, I knew they would find me". [Ahl et al., participant quotation]

"The stress and pain began to ease when I knew that the ambulance was on its way" [Ahl et al., participant quotation]

Also closely linked with reassurance and uncertainty is the descriptive theme of compassion. Codes within the compassion theme sometimes occurred alongside codes within the practical 'ambulance as transport' theme, but in a manner that suggests at least some patients do see these two roles as distinct:

"Well, they took care of me and put me on a stretcher, they checked the whole time that I was all right so it sure was more care than transport". [Ahl et al., participant quotation]

For peer review only - http://bmjopen.bmj.com/site/about/guidelines.xhtml

The perceived absence of compassion when calling for an ambulance was also noteworthy, impacting upon the overall experience:

“He didn’t care. It was just when I was speaking to him he wasn’t sort of listening”. [Togher et al., participant quotation]

Such codes occurred commonly with examples of clear mismatch between the ambulance clinician’s assessment and the patient’s assessment of medical need. In such circumstances the emotional function of the ambulance service was not fulfilled, even if the medical function was.

The Process Domain

Central to this analysis is the third analytical theme of the ‘processes’ involved in balancing the practical domain with the emotional domain. This analytical theme relates the emotional aspects of seeking urgent help with the practicalities of obtaining and receiving ambulance treatment, by describing processes central to help-seeking and decision-making in this context. These ‘process’ descriptive themes were grouped into three: being influenced in the decision-making by others, being empowered to take responsibility for one’s health, and taking or handing over control of the situation. This analysis demonstrated the frequent juxtaposition – even tension – of some of the themes related to practicalities with themes related to emotional responses.

A strong descriptive theme running through the analysis of the process domain was the idea of patients achieving some sense of control over a perceived urgent or distressing situation by phoning an ambulance:

For peer review only - http://bmjopen.bmj.com/site/about/guidelines.xhtml

"When patients phone for an ambulance, they hope to gain control of the experienced emergency situation based on trust and the expectation that they will be assisted… which implies an ethical demand." [Rantala et al., author interpretation]

"It feels good once you have made the decision and phoned for an ambulance. They know that something is wrong… now I don't have to take further responsibility" [Ahl et al., participant quotation]

The approach of decisively 'taking control' of the situation appears to be used by some patients to bring about a definitive resolution to an otherwise intolerable situation, by accessing ambulance treatment. Contrastingly, some patients appear much more comfortable with handing over control of a situation they don't fully understand to the ambulance service, thereby exonerating themselves of responsibility for managing it. This concept of 'responsibility' appears quite complex. Linked with the emotional theme of reassurance is a need to also be 'reassured' that the decision to call an ambulance was correct – to seek legitimisation for using a finite (even scarce) resource for one's own needs. When callers 'hand over' this practical element of the decision-making by calling the emergency number, they appear to feel emotionally more comfortable with the outcome of an ambulance attending, as it is not directly they who made the decision to send one. Rather it is the *system* that has decided that an ambulance is necessary, and is responsible for the outcome – there appears a degree of conceptual disconnect between calling for an ambulance and receiving one.

It is also clear how powerful the influence of others (particularly family) can also be on this particular aspect of effectively 'handing over' the decision-making process:

For peer review only - http://bmjopen.bmj.com/site/about/guidelines.xhtml

"It was my wife who was very concerned and wanted to make the call". [Rantala et al., participant quotation]

A particularly important aspect of this descriptive theme is that the 'influencer' doesn't even necessarily have to be present at the time of the need for treatment for their views to form part of the decision-making:

"I could just hear my daughter saying "oh mum, why didn't you get the ambulance out?" even though I didn't think I needed it." [Booker et al., participant quotation]

Ambulance crews also have an ability to exert influence on the patients' decision-making, particularly around whether hospitalisation is necessary or not. Particularly noteworthy in the analysis is that the process of exerting this influence – whilst often conveyed in very practical terms (such as the likely lengthy waiting around in hospital for a bed to be available) – has the ability to produce a strong emotional response. This emotional response can often be one of empowerment and taking responsibility, through mutual respect, partnership and shared decision-making:

"[Paramedics] also talked a great deal about the possibility of persuading people to stay at home when hospital treatment was not necessary" [Porter et al., author interpretation]

"So I was respected as a full partner in the conversation. It was a very nice experience" [Rantala et al., participant quotation]

But the process of influencing can also result in negative emotional responses such as guilt, which may even have direct consequences on the patient's choice of care in the future. Commonly the codes associated with negative influences occurred in encounters that were being labelled as 'inappropriate':

For peer review only

For peer review only - http://bmjopen.bmj.com/site/about/guidelines.xhtml

"Afterwards I felt very guilty about what happened and why I had done it. I just hope I don't have to call them again." [Rantala et al., participant quotation]

"One feels so worthless… they just don't believe me!" [Rantala et al., participant quotation]

For peer review only

For peer review only - http://bmjopen.bmj.com/site/about/guidelines.xhtml

DISCUSSION

This qualitative synthesis suggests that the decision-making processes involved in calling for and receiving ambulance treatment for a ‘primary care sensitive’ problem are shaped by a combination of emotional and practical factors. Often, the ambulance service contact functions to fulfil *both* emotional and practical needs in the caller. Regardless of the actual severity of the precipitating clinical condition, both of these groups of factors play an important role in choosing (and valuing) ambulance-delivered care. This synthesis proposes that three key processes are involved in combining emotional and practical factors: handing over of control and responsibility, the influencing by important others, and empowerment to take responsibility for one’s own health. Informal carers, relatives and health professionals can all be ‘influencers’, and do not even necessarily have to be physically present for their ‘influence’ to be considered by a patient.

There is currently substantial discussion in the literature about the need to reduce so-termed ‘unnecessary’ or ‘inappropriate’ ambulance use. However, this synthesis supports previous findings [5] that suggest the concept of ‘inappropriate’ ambulance use is far more complex than the fundamental medical condition that a patient receives treatment for. Indeed, this analysis suggests there is a strong association with negative emotional responses when callers believe they are being labelled as ‘inappropriate’, which may have implications for their future attempts to access care. The themes from this review suggest that urgent care triage systems need to be sensitive to both the perceived emotional and practical drivers of a call for help, but importantly need to recognise how this may be shaped by carers and relatives. The ambulance service contact is sometimes therapeutic in itself. It appears that the components of the treatment contact that patients value are those that support their own ability to ‘process’ both the emotional and practical challenges they are

For peer review only - http://bmjopen.bmj.com/site/about/guidelines.xhtml

For peer review only

presented with by their health condition, and enable them to find a comfortable level of control of the situation and possible outcomes.

Conclusions

Supporting patients and their relatives to access the “right advice in the right place, first time” has underpinned UK national urgent care policy for nearly a decade [26], but is hampered by an incomplete understanding of how patients conceptualise urgency. This synthesis builds on recent work in the emergency department setting, that suggested patients define situations worthy of emergency health resources according to socio-emotional factors rather than purely the symptoms or physiology underlying their illness.[27] This synthesis also suggest that the ‘organisation incentives’ and ‘secondary benefits’ of the health system referred to in the classic sociological models of illness and help-seeking [8] may also exist when choosing ambulance care via the processes of legitimisation and, perhaps somewhat paradoxically, empowerment. Previous work has acknowledged how practical issues such as rurality and remoteness,(e.g.[28,29]) perceptions of out of hours primary care,(e.g.[30,31]) and views of telephone advice services [32] shape decision-making. This synthesis highlights how importantly patients regard the views of those around them, and how emotionally conflicted some patients can be about their care choices.

Practice Implications

This synthesis is the result of a comprehensive, systematic literature search in accordance with a prospectively published protocol. Although the resulting synthesis is based on a relatively small number of studies meeting the inclusion criteria, this reflects a relatively under-researched area of interest. The majority of included

For peer review only - http://bmjopen.bmj.com/site/about/guidelines.xhtml

studies are from UK-based ambulance services, and all were undertaken in a European system with a primary health care model. As such, there may be some limitations in applicability to contrasting ambulance systems. Additionally, all reported studies were based on adult populations, limiting the conclusions that can be drawn for paediatric populations. Despite these limitations, this study is, to the authors' knowledge, the first synthesis of qualitative evidence combining patients', carers' and health professionals' perspectives on the use of ambulances for lower-acuity conditions. Future work needs to explore how urgent care systems can be sensitive and responsive to the emotional component of the help-seeking process, whilst still providing the practical assistance that patients and carers require. This necessarily will involve a balanced approach that empowers urgent healthcare providers to tailor the format of the response accordingly.

References

For peer review only - http://bmjopen.bmj.com/site/about/guidelines.xhtml

[1] Pickering A, Mason S, Turner J, et al. A comparative review of ambulance service best practice. Office of the Strategic Health Authorities. http://www.nhsimas.nhs.uk/fileadmin/Files/IST/ESR_A_comparative_review_of_intrnational_ambulance_service_best_practice.pdf Access Date: March 2017

[2] Department of Health, Taking healthcare to the patient: transforming NHS Ambulance Services. http://ircp.info/Portals/11/Future/NHS%20EMS%20Policy%Recommendation2005.pdf Access Date: March 2017

[3] Department of Health, Tackling demand together. A toolkit for improving urgent and emergency care pathways by understanding increases in 999 demand. http://www.dh.gov.uk/prod_consum_dh/groups/dh_digitalassets/documents/digitalasset/dh_106924.pdf Access Date: March 2017

[4] NHS England, High quality care for all, now and for future generations: transforming urgent and emergency care services in England. The evidence base from the urgent and emergency care review. http://www.england.nhs.uk/wp-content/uploads/2013/review Access Date: March 2017

[5] Booker MJ, Shaw ARG, Purdy S. Why do patients with 'primary care sensitive' problems access ambulance services? A systematic mapping review of the literature. *BMJ Open* 2015;5:e007726.

[6] Durand AC, Palazzolo S, Tanti-Hardouin N, et al. Nonurgent patients in emergency departments: rational or irresponsible consumers? Perceptions of professionals and patients. *BMC Research Notes* 2012;5:525.

For peer review only - http://bmjopen.bmj.com/site/about/guidelines.xhtml

[7] Booker MJ, Simmonds RL, Purdy S. Patients who call ambulance for primary care problems: a qualitative study of the decision-making process. *Emerg Med J* 2014;31:448-52.

[8] Mechanic D. Sociological Dimensions of Illness Behavior. *Soc Sci Med* 1995;41:1207–16.

[9] Hunter C, Chew-Graham C, Langer S, et al. A qualitative study of patient choices in using emergency health care for long-term conditions: the importance of candidacy and recursivity. *Patient Educ Couns* 2013;93:335-41.

[10] Langer S, Chew-Graham C, Hunter C, et al. Why do patients with long-term conditions use unscheduled care? A qualitative literature review. *Health and Social Care in the Community* 2013;21:339-51.

[11] Thomas J, Harden A. Methods for the thematic synthesis of qualitative research in systematic reviews. *BMC Medical Research Methodology* 2008;8:45.

[12] Strauss AL, Corbin J. Basics of qualitative research: grounded theory procedures and techniques. Newbury Park, CA: Sage 1990

[13] Tong A, Flemming K, McInnes E, et al. Enhancing transparency in reporting the synthesis of qualitative research: ENTREQ. *BMC Med Res Methodol* 2012;12:181.

[14] Campbell R, Pound R, Pope C, et al. Evaluating meta-ethnography: a synthesis of qualitative research on lay experiences of diabetes and diabetes care. *Soc Sci Med* 2003;56:671-84.

For peer review only

For peer review only - http://bmjopen.bmj.com/site/about/guidelines.xhtml

[15] Dixon-Woods M, Cavers D, Agarwal S, et al. Conducting a critical interpretive synthesis of the literature on access to healthcare by vulnerable groups. *BMC Med Res Methodol* 2006;6:35-47.

[16] Seale C. Quality in qualitative research. *Qual Inq* 1999;5:465-78.

[17] Spencer L, Richie J, Lewis J, et al. Quality in qualitative evaluation: a framework for assessing research evidence. London: Cabinet Office 2003.

[18] Walsh D, Downe S. Appraising the quality of qualitative research. *Midwifery* 2006;22:108-19.

[19] Critical Appraisal Skills Programme (CASP) CASP Checklists. Oxford: UK. http://www.casp-uk.net/#!casp-tools-checklists/c18f8 Access Date March 2017

[20] Downe S. Metasynthesis: a guide to knitting smoke. *Evidence Based Midwifery* 2008;6:4-8.

[21] Ahl C, Nystrom M, Jansson L. Making up one's mind: patients' experiences of calling an ambulance. *Accid Emerg Nurs* 2006;14:11-19.

[22] Campbel NC, Iversen L, Fermer J. A qualitative study in rural and urban areas on whether – and how – to consult during routine and out of hours. *BMC Family Practice* 2006;7:26.

[23] Rantala A, Ekwall A, Forsberg A. The meaning of being triaged to non-emergency ambulance care as experienced by patients. *Int Emerg Nurs* 2016;25:65-70.

For peer review only

For peer review only - http://bmjopen.bmj.com/site/about/guidelines.xhtml

[24] Porter A, Snooks H, Youren A, et al. 'Should I stay or should I go?' Deciding whether to go to hospital after a 999 call. *J Health Serv Res Policy*. 2007;S1:32-8.

[25] Togher FJ, O'Cathain A, Phung VH, et al. Reassurance as a key outcome valued by emergency ambulance service users: a qualitative interview study. *Health Expectations* 2015;18:2951-61.

[26] Department of Health. NHS next stage review: Our vision for primary and community care. http://www.nhshistory.net/dhvisionphc.pdf Access Date March 2017

[27] Morgans A, Burges SJ. What is a health emergency? The difference in definition and understanding between patients and health professionals. *Australian Health Review* 2011;35:284-9.

[28] Farmer J, Iversen L, Campbell NC, et al. Rural/urban differences in accounts of patients' initial decisions to consult primary care. *Health & Place* 2006;12:210-21.

[29] Turnbull J, Pope C, Martin D, et al. Do telephones overcome geographical barriers to general practice out-of-hours services? Mixed-methods study of parents with young children. *Journal of Health Services Research and Policy* 2010;15:21-7.

[30] Egbunike JN, Shaw C, Bale S, et al. Understanding patient experience of out-of-hours general practitioner services in South Wales: a qualitative study. *Emerg Med J* 2008;25:649-54.

[31] Foster J, Dale J, Jessopp L. A qualitative study of older people's views of out-of-hours services. *BJGP* 2001;51:719-23.

For peer review only - http://bmjopen.bmj.com/site/about/guidelines.xhtml

[32] O'Cathain A, Knowles E, Turner J, et al. Acceptability of NHS 111 the telephone service for urgent health care: cross sectional postal survey of users' views. *Family Practice* 2014;31:193-200.

For peer review only

For peer review only - http://bmjopen.bmj.com/site/about/guidelines.xhtml

CONTRIBUTORS

MJB is the main author involved in all stages of the design, analysis and write-up. ARGS and SP contributed to the methodological approach, data extraction, verification and analysis through regular research meetings. ARGS and SP contributed significant input to the written paper. All authors have approved the final version of the paper ahead of publication.

ACKNOWLEDGMENTS

None.

COMPETING INTERESTS

None declared.

FUNDING

MJB is funded by a National Institute for Health Research Doctoral Research Fellowship. This paper presents independent research funded by the National Institute for Health Research (NIHR). The views expressed are those of the author(s) and not necessarily those of the NIHR, the NHS or the Department of Health.

DATA SHARING STATEMENT

No additional data are available.

For peer review only

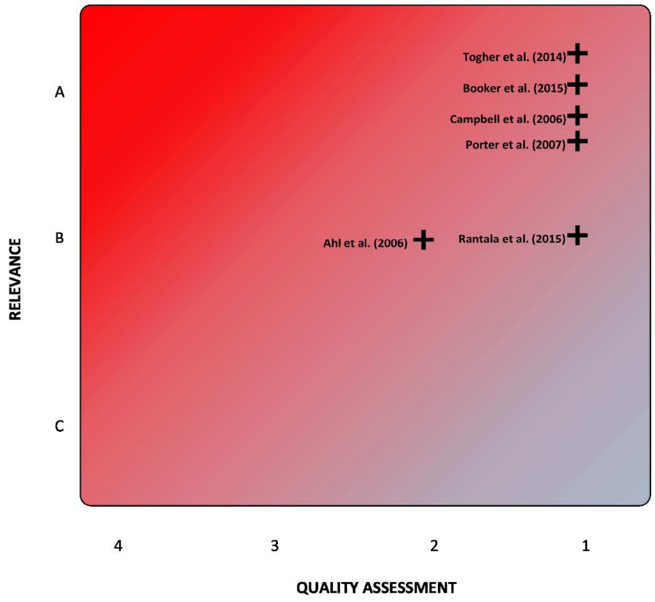

'Quality-Relevance plot' of included studies.

209x297mm (300 x 300 DPI)

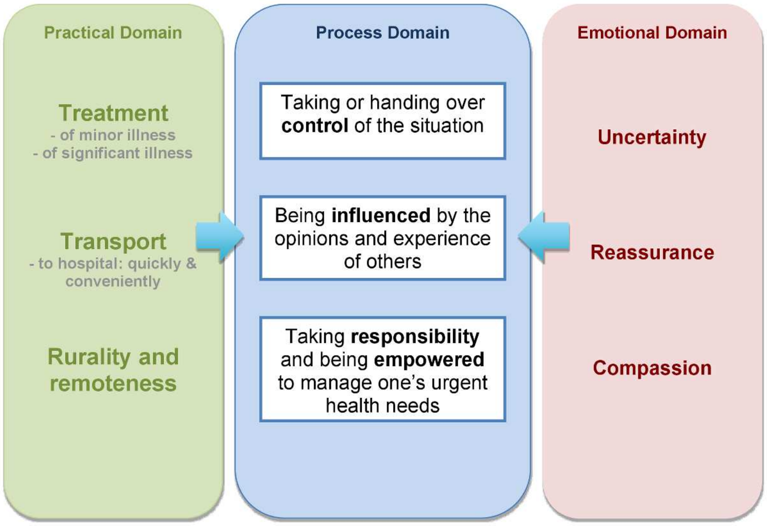

Relationships between analytical themes

209x297mm (300 x 300 DPI)

BMJ Open

Seeking ambulance treatment for 'primary care' problems: a qualitative systematic review of patient, carer and professional perspectives.

Journal:	*BMJ Open*
Manuscript ID	bmjopen-2017-016832.R1
Article Type:	Research
Date Submitted by the Author:	06-Jun-2017
Complete List of Authors:	Booker, Matthew; University of Bristol, School of Social and Community Medicine<br>Purdy, Sarah; University of Bristol, Centre for Academic Primary Care<br>Shaw, Ali; University of Bristol, School of Social and Community Medicine
**Primary Subject Heading**:	Emergency medicine
Secondary Subject Heading:	General practice / Family practice, Health services research, Qualitative research
Keywords:	Ambulance, PRIMARY CARE, Urgent Care, Decision-making

SCHOLARONE™
Manuscripts

For peer review only

Seeking ambulance treatment for 'primary care' problems: a qualitative systematic review of patient, carer and professional perspectives.

Matthew J Booker [a]

Sarah Purdy [a]

Alison R G Shaw [a]

Author Affiliations: [a] Centre for Academic Primary Care, School of Social & Community Medicine, University of Bristol, Bristol, UK

Corresponding author at:

Matthew J Booker, Office 1.07, School of Social and Community Medicine, University of Bristol, Canynge Hall, Whatley Road, Bristol, UK BS8 2PS

Matthew.Booker@Bristol.ac.uk

+44 (0)117 9287305

Word Count: 4557

For peer review only

For peer review only - http://bmjopen.bmj.com/site/about/guidelines.xhtml

ABSTRACT

Objectives: To understand the reasons behind, and experience of, seeking and receiving emergency ambulance treatment for a ‘primary care sensitive’ condition.

Design: A comprehensive, qualitative systematic review. MEDLINE, EMBASE, PsychInfo, CINAHL, HMIS, HMIC, OpenSigle, EThOS and DART databases were systematically searched for studies exploring patient, carer or healthcare professional interactions with ambulance services for ‘primary care sensitive’ problems. Studies using wholly qualitative approaches, or mixed-methods studies with substantial use of qualitative techniques in both the methods *and* analysis sections were included. An analytical thematic synthesis was undertaken, using a line-by-line qualitative coding method, and a hierarchical inductive approach.

Results: Of 1458 initial results, 33 studies met the first level (relevance) inclusion criteria, and 6 studies met the second level (methodology and quality) criteria. The analysis suggests that patients define situations worthy of ‘emergency’ ambulance use according to complex socio-emotional factors, as well as experienced physical symptoms. There can be a mismatch between how patients and professionals define ‘emergency’ situations. Deciding to call an ambulance is a process shaped by practical considerations and a strong emotional component, which can be influenced by the views of caregivers. Sometimes the value of a contact with the ambulance service is principally in managing this emotional component. Patients often wish to hand over responsibility for decisions when experiencing a perceived emergency. Feeling empowered to take control of a situation is a highly valued aspect of ambulance care.

For peer review only - http://bmjopen.bmj.com/site/about/guidelines.xhtml

Conclusions: When responding to a request for 'emergency' help for a low-acuity condition, urgent-care services need to be sensitive to how the patient's emotional and practical perception of the situation may have shaped their decision-making, and the influence that carers may have had on the process. There may be novel ways to deliver some of the valued aspects of urgent care, more geared to the resource-limited environment.

Keywords

Ambulance; Urgent Care; Primary Care; Decision-making;

STRENGTHS AND LIMITATIONS OF THIS STUDY

- This evidence synthesis expressly focuses on the experiences of providing and receiving ambulance-care for 'primary care sensitive' clinical problems, which are forming an increasing part of pre-hospital care workload.
- The methods of qualitative systematic review build on previous literature mapping work, to offer a more nuanced appreciation of the complexities experienced by patients, carers and professionals in seeking ambulance treatment.
- The majority of qualitative evidence is drawn from ambulance systems in more economically developed countries with an established primary care model, and in adult populations.

For peer review only

INTRODUCTION

Despite origins as services for those with acute medical emergencies and injuries,[1] the majority of contacts with ambulance organisations are no longer for serious immediately life-threatening conditions.[2] Calls to the emergency ambulance service in the UK have been rising over recent years at 7% per annum,[3] and are increasingly for conditions that could potentially be managed by a primary care provider.[4] The proportion of ambulance calls for mental health conditions and social situations are also rising for reasons that are poorly understood.[4]

A recent systematic mapping review by the authors identified a number of factors that may be associated with ambulance use for problems that could be managed in primary care, including markers of deprivation, minority status and certain social circumstances.[5] Much of the previous literature exploring help-seeking for such problems in the context of ambulance use has focussed on the so-termed 'inappropriate' use of the service, usually defined from the health care professional perspective. However, defining use in this way neglects to appreciate the complex processes that are likely to underlie help-seeking behaviours and attitudes in these user groups. Indeed, healthcare professionals themselves lack consensus as to what constitutes 'inappropriate' use of emergency healthcare.[6] There is increasing acceptance that labelling such ambulance services use as inappropriate fails to recognise the context of the request for assistance, and is unhelpful for developing practical solutions. Additionally, making the decision to call an ambulance is not always the 'easy' option for many service users, as previous qualitative research by the authors [7] suggests that many emergency calls are preceded by a substantial degree of internal conflict on the part of the caller about the best course of action.

For peer review only - http://bmjopen.bmj.com/site/about/guidelines.xhtml

An established body of sociological research has sought to unpick what underpins illness behaviour and help-seeking in general. The more established models of illness and help-seeking acknowledge a complex interplay between biological predisposition to illness, experienced symptomatology, learned behaviour patterns, attributional predispositions, situational influences, and the organisational incentives and secondary benefits of the health care system itself.[8] Recent work examines how help-seeking and decision-making models may apply to some urgent care settings, such a GP Out-of-Hours [9] and Emergency Departments,[10] but there has been a lack of attention to how patients and carers conceptualise the need for ambulance care specifically. There is very limited understanding of how help-seeking models apply to situations where the request for ambulance care is for a problem that is potentially manageable in a primary care setting.

This paper, therefore, seeks to provide a more detailed and nuanced account of the experience of seeking and receiving ambulance treatment for ‘primary care sensitive’ conditions, by synthesising relevant data from qualitative studies of patients, carers and professionals.

For peer review only

For peer review only - http://bmjopen.bmj.com/site/about/guidelines.xhtml

METHODS

An electronic systematic literature search was undertaken on the following databases: MEDLINE, EMBASE, PsychInfo, CINAHL, Health Management Information Consortium and Health Management Information Service. Google Scholar and Web of Science searches were undertaken to identify reports not captured by the above. Additional searches of OpenSigle, EThOS and DART databases were undertaken. The comprehensive systematic strategy was complemented by hand searches of key journals. This literature search formed the basis of a systematic literature mapping exercise,[5] the protocol of which has been published on the PROSPERO register (reference CRD42014009108). Search terms were developed iteratively by consensus discussion amongst the research team and a medical subject librarian. An example search strategy is included as Appendix 1. Initial searches were run for papers published between January 1980 and June 2014, with further update searches covering the period June 2014 to January 2017.

Inclusion and Exclusion Criteria

The inclusion criteria for the systematic mapping review [5] were articles published in the English language, reporting the findings of research exploring patient, carer, healthcare professional or health service management perspectives and experiences of interaction with ambulance services for ‘primary care sensitive’ problems. The definition of a ‘primary care sensitive’ condition could be from any perspective, assigned prospectively or retrospectively. A ‘primary care sensitive’ contact could include explicit reference to terms related to primary care or family medicine, or could be defined by reference to a comprehensive list of indicator conditions developed in conjunction with a medical subject librarian. Studies that reported on any stage of the

For peer review only - http://bmjopen.bmj.com/site/about/guidelines.xhtml

interaction (from telephone call to treatment or transport) were of interest. Studies reporting on routine primary care only were excluded.

For this qualitative systematic review, further inclusion criteria were applied to identify papers reporting qualitative research. Papers were included if the data collection and analysis were wholly qualitative. Mixed-methods papers were included if they reported identifiable components that had used qualitative data collection and analysis methods. Papers were screened by two researchers (MB, AS) independently, with all excluded papers further reviewed by all researchers to ensure agreement.

Extracting, Coding and Analysing the Data

A thematic synthesis was undertaken, following the approach described by Thomas and Harden.[11] All verbatim text in the 'results' and 'discussion' sections (or equivalent) of papers was treated as data for the purposes of this analysis. Data were loaded electronically into NVivo software (version 11), and a process of line-by-line open coding undertaken. Results, which often included quotations or descriptions of themes, were coded alongside authors' interpretive statements in the discussion section. The synthesis took the form of a three-level inductive thematic analysis: open line-by-line coding of the primary studies, organisation into groups of descriptive themes, and finally the development of overarching analytical themes.[12] The ENTREQ statement was followed throughout the data collection, appraisal and synthesis.[13]

There are a range of methodological approaches to handling and analysing data as part of a qualitative synthesis,[13] including meta-theoretical and meta-ethnographic approaches that draw upon grounded theory and line-of-argument principles to

For peer review only - http://bmjopen.bmj.com/site/about/guidelines.xhtml

synthesise ‘key concepts’ (e.g.[14]) and critical interpretive methods resulting in synthetic constructs. (e.g.[15]) The line-by-line thematic synthesis approach [11] was used in this analysis. With philosophical foundations in critical realism, this approach permits a holistic understanding of the described phenomena through a more flexible way that ‘free’ first levels codes can be applied to various types of source data in the included studies. Table 1 provides example extracts from the analysis to illustrate this process of coding ‘up’ from free codes, to descriptive then analytical themes. MB led on this process with regular research meetings between all authors to verify coding and agree themes by consensus.

Original Text	Free Codes	Descriptive Themes	Analytical Themes
That’s the worst of staying out here in the wilds out here because they speak about this nine minutes and stuff o’ this kind, but that is impossible staying oot here, like	3.45 Difficult/impossible for ambulances to arrive quickly/meet response time targets in geographically remote areas.<br>3.46 Acknowledgement that living in rural area necessarily has (disadvantage) of time delay in getting emergency care.	RURALITY AND REMOTENESS: There are consequences of living in a rural or remote area in terms of how quickly ambulance care can be accessed. Patients recognise and even accept this constraint, and adapt their expectations and the way they decide to physically access care, and the way in which they get to the location of treatment.	**Practical Domain** – whatever the perceived health need may be, and regardless of who may be involved assessing it, there are physical practicalities that sometimes override all other aspects of the decision-making process and take precedence. These can be related to geography and space, access to modes of transport, physical limitations of an individual’s capability to care for themselves or the perceived need for immediate, expert care that can only be provided by ambulance staff.
If it was very severe, I would get my husband… to drive me to the hospital	3.72 Very severe illness requires hospital treatment.<br>3.73 Assistance from relative required to access appropriate care.<br>3.74 Decision to drive/make own way to hospital instead of call and wait for ambulance.		
I could have gone in my car… I would have made a lot of work for my relatives, which I don’t think is right.	2.92 Health condition such that could have gone by car, but active decision not to due to convenience.<br>2.93 Assistance from relative required to access care<br>2.94 Reluctance to inconvenience relatives as a result of own illness.	TRANSPORT: role of seeing the ambulance service as a transport when other options are discounted as unsuitable or inconvenient, even when they may be entirely suitable as the clinical condition is relatively minor.	**Process Domain**: **being influenced by others** – perception of whether one would be a burden on one’s relatives shapes the decision making process about how to access urgent treatment (subtle or ‘invisible’ influence of relatives).

For peer review only - http://bmjopen.bmj.com/site/about/guidelines.xhtml

Table 1: Example data extracts demonstrating the hierarchical coding process

Assessment of Quality

The assessment of quality in qualitative syntheses is much debated and there is little consensus about whether to do quality appraisal and how to do it.[11] Some qualitative methodologists argue that quality should not or cannot be meaningfully measured at all,[16,17] particularly if the synthesis aims to include all relevant existing data, however collected or reported. The authors of this synthesis take the view that while relevance of a paper to the review may outweigh reporting quality, it is helpful to attempt to assess quality in a consistent and transparent manner, to allow the reader to make judgements about how particular papers have contributed to a synthesis. In this synthesis, whilst studies were not excluded on the basis of a quality assessment alone, recognition of the quality limitations of included papers enabled a sensitivity analysis to be performed as described below.

A number of tools have been developed to assist in the systematic appraisal of quality in qualitative research.[18] Many of these are limited to informing the inclusion/exclusion process, and some have been criticised for blending different aspects of quality assessment under too few headings. The Critical Appraisal Skills Programme (CASP) checklist [19] is often utilised for quality assessment in qualitative syntheses, prompting assessment of a paper against a number of items related to the purpose, design, conduct and reporting of qualitative research. A modified CASP checklist was used in this synthesis to assess included papers under a number of headings: overall appropriateness of the qualitative methodology, credibility, transferability, dependability, and confirmability, including detail of the reporting. Whilst there is considerable debate about assigning numerical ‘scores’ to such quality appraisals, the synthesis team adopted the four-tiered quality summary

For peer review only

score described by Downe [20] to report the output of this quality assessment in a transparent manner. The final quality score, described in Table 2, was assigned by consensus amongst the research team following the appraisal process described above.

Score	***Description***
1	No or few flaws: credibility, transferability, dependability and confirmability is high.
2	Some flaws: unlikely to affect the credibility, transferability, dependability or confirmability.
3	Some flaws, which may affect the credibility, transferability, dependability, and/or confirmability
4	Significant flaws, which are very likely to affect the credibility, transferability, dependability and/or confirmability

Table 2: Quality summary scores for qualitative studies (adapted from Downe [20])

To enable judgement about how individual papers of varying reporting quality had contributed to the synthesis, a form of sensitivity analysis was undertaken as described by Thomas and Harden,[11] whereby the themes arising from each of the included papers were supressed from the analysis. This was achieved by removing all coded data from the NVivo dataset, paper by paper, and comparing the model against the full analysis. There was no substantial impact on the top-level conceptual model as a result of this, indicating that the final model was not disproportionately shaped by studies of any particular quality.

For peer review only - http://bmjopen.bmj.com/site/about/guidelines.xhtml

The Quality-Relevance (Q-R) Plot

Assessing relevance of qualitative studies in a synthesis is also an area of some debate, and often includes assessment of some parameters that could arguably overlap with measurements of quality.[16] For the purposes of this qualitative synthesis, the concept of relevance is defined as applicability of the study's findings to the authors' health system and infrastructure, namely a (broadly) nationally homogenous ambulance service operating within a health infrastructure with an established primary-care model. As such, relevance was assessed – again by consensus – into one of three categories, as summarised in Table 3:

A	Concerning UK Ambulance Services and/or UK 'Primary Care' Model
B	Concerning Westernised (but non-UK) Ambulance Service and/or non-UK 'Primary Care' based model of healthcare
C	Concerning non-Westernised Ambulance Service and/or non-Primary Care based model of healthcare.

Table 3: Categorisation of study relevance

In order to visualise the spread of included studies by both quality and relevance, the authors developed the concept of the Quality-Relevance (Q-R) plot (Figure 1).

Figure 1: 'Quality-relevance plot' of included studies

For peer review only - http://bmjopen.bmj.com/site/about/guidelines.xhtml
For peer review only

RESULTS

Search results

The literature search identified 1458 references (n=1424 during the initial search, and n=34 in the update search). After duplicate suppression and removal of irrelevant and incorrectly cited documents, first level screening against the inclusion/exclusion criteria resulted in 33 studies remaining (n=31 from the initial search and n=2 from the update search). Second level screening for qualitative methods content resulted in 6 papers (n=5 from the initial and n=1 from the update searches) meeting full inclusion criteria for the qualitative synthesis. The characteristics of these studies are summarised in Table 4. Figure 1 summarises the authors' assessment of quality and relevance as described above.

Paper	Year	Setting	Sample	Study Methodology
Booker et al. [7]	2013	UK Ambulance Service	16 adult participants (patients and carers)	Qualitative semi-structured interview study; thematic analysis
Ahl C et al. [21]	2006	Swedish Ambulance Service	20 adult participants (patients)	Qualitative interview study, content analysis.
Campbell et al. [22]	2006	Scottish Primary Care	78 adult participants (patients)	Qualitative semi-structured interviews and focus groups, inductive thematic analysis
Rantala et al.	2015	Swedish Ambulance	12 adult participants	Qualitative open-ended interviews, inductive

For peer review only - http://bmjopen.bmj.com/site/about/guidelines.xhtml

[23]		Service	(patients)	phenomenological hermeneutic analysis
Porter et al. [24]	2007	UK Ambulance Service	25 adult participants (paramedics)	Qualitative focus group study; thematic analysis
Togher et al. [25]	2014	UK Ambulance Service	30 (22 patients, 8 spouses)	Qualitative interview study, thematic analysis and mapping.

Table 4: Characteristics of the studies included in the thematic synthesis

From Open Codes to Descriptive Themes

Following the process of free coding, a total of 23 descriptive themes were developed iteratively by repeated cycles of reductive grouping of codes until no additional discrete categories were required to fully describe the free-code dataset. As both verbatim primary data transcriptions and authors' interpretations were included as 'data' in this exercise, these descriptive themes already have a degree of interpretation included in them. These descriptive themes were then organised into nine, related descriptive theme groups. The relationship is shown in Table 5.

For peer review only - http://bmjopen.bmj.com/site/about/guidelines.xhtml

Receiving treatment for a complaint that the caller knows is minor or non-serious.	Needing and receiving **treatment** urgently
Receiving immediate, life saving treatment for a condition the caller suspects is serious or life-threatening.	
Skillset and capabilities of the ambulance staff providing treatment	
Using the ambulance as a means of quick transport to hospital (when there is a medical need)	The **transport** role of the ambulance service
Using the ambulance as a 'convenient' mechanism of transport to hospital (with or without medical need)	
The impact of rurality and geographic remoteness on the care available when needed	**Rurality and remoteness** and the impact on receiving ambulance care
The impact or rurality and geographic remoteness on personal expectations and choices about accessing care	
Patient uncertainty about the seriousness of their health conditions, and whether there is urgent medical need	**Uncertainty** about own health and the choice for ambulance care
Patient uncertainty about how to access the most appropriate health care service	
Patient uncertainty about the impact of their actions on others	
Uncertainty about making a decision on behalf of someone else (relative or friend)	
Need for personal reassurance that there is no underlying serious health problems	**Reassurance** about the absence of serious health problems
Need for reassurance that there is a legitimate need to access ambulance care	
Providing information to medical professionals to allow them to make appropriate decisions	Taking or handing over **control** of the situation
Taking decisive action to manage an intolerable circumstance	
Compassion provided by ambulance staff (in comparison to other healthcare groups)	Experience of **compassion** during contact with the ambulance service
Compassion of friends and relatives in times of illness when calling an ambulance	
Influence of others (friends and relatives) when asked for their opinion	Being **influenced** by the opinions and experience of others about ambulance care
Influence of others (friends and relatives) through their perceived opinions, even though the person may not present or directly consulted	
The need to take responsibility for one's own healthcare	Taking **responsibility** and being **empowered** to manage one's urgent health needs
The need to take responsibility for the healthcare of another (friend or relative)	
Empowerment to manage own healthcare	
Empowerment to access ambulance care appropriately	

Table 5: Relationship between descriptive themes and thematic groups

For peer review only - http://bmjopen.bmj.com/site/about/guidelines.xhtml

Development of Analytical Themes

By analysing patterns in the free codes and descriptive themes within and across t the nine thematic groups, a number of cross-relationships between groups were identified. Through a process of comparing the theme groups and their constituent descriptive themes, three overarching analytical themes were identified. These analytical themes conceptualise 'domains' of experience that are shared by all involved in the event of ambulance treatment for primary care sensitive conditions: practical domain, emotional domain and process domain.

The **practical** domain contains descriptive themes relating to the practicalities of needing and receiving treatment, including themes associated with access issues and transport. The **emotional** domain brings together descriptive themes concerning the emotions involved in needing, calling for, receiving and providing ambulance treatment. The **process** domain is a conceptually distinct and central domain, referring to the processes by which patients, carers and professionals try to integrate the practical needs and limitations of the situation with the emotional challenges associated with illness and help-seeking. Figure 2 summarises how the three analytical themes are related.

Figure 2: Relationships between analytical themes

For peer review only

For peer review only - http://bmjopen.bmj.com/site/about/guidelines.xhtml

The practical domain

This analytical theme unites descriptive themes relating to the practicalities of asking for or providing ambulance care. Whilst the practical aspects (including logistic difficulties and convenience) may not always be the first consideration, many of the themes include some aspect of patients and carers ‘weighing up’ how practical the use of the ambulance service (or alternatives) are for their perceived needs.

In many instances, patients appear to know, broadly, what is wrong with them and what treatment is required, but do not appreciate that the problem is out-of-scope of the ambulance service. Sometimes patients appear to genuinely expect the ambulance service to treat minor ailments and injuries in preference to GP services – a belief that appears rooted in misunderstanding of how urgent care services are structured. In these circumstances, the call to the ambulance service is made in ‘good faith’ that this is the right way to access treatment. Sometimes, however, patients acknowledge that the condition is not life threatening, but feel that there are genuine practical issues that would prevent appropriate treatment elsewhere, in what they see as a logical and reasoned attempt to arrive at the most sensible outcome:

“The doctor would find it very difficult to do anything useful, I think, just with me describing the symptoms of a water infection over the telephone”. [Booker et al., participant quotation]

“The doctor on the phone said [it may be] early appendicitis. You are bound to need blood tests and scans and stuff… so I just called the ambulance to get straight up to hospital.” [Booker et al., participant quotation]

The logic behind these decisions is not fundamentally unsound, but there is a lack of understanding of the implications of these access choices on the wider system.

For peer review only - http://bmjopen.bmj.com/site/about/guidelines.xhtml

Practical issues appear to be viewed in differing ways, with often minor logistic barriers being used as a justification for using ambulance services. The theme of the ambulance service fulfilling a transport role is one such example, with the idea of getting to hospital more quickly or more easily being central. There are examples of when patients articulate that ambulance transport is more convenient, if not strictly a necessity:

"I could have gone in my car… I would have made a lot of work for my relatives, which I don't think is right". [Ahl et al., participant quotation]

Thus, while not always overt in the primary data, there were connections between data within the descriptive theme about 'convenience' of ambulance service use and data within the descriptive theme relating to perceived impact upon others around the patient. Thus, 'convenience' extends to include patients not wishing to be a burden on their carers and relatives.

Convenience can also be viewed from the perspective of physical and geographic isolation. Being geographically remote was something that some patients acknowledged as impacting upon what kind of service they might expect from the ambulance:

"That's the worst of staying out here in the wilds out here because they speak about this nine minutes and stuff o' this kind, but that is impossible staying oot here, like." [Campbell et al., participant quotation]

This differing geography resulted in those living in urban areas taking a notably different course of action to those living in rural areas, seemingly as a result of the

For peer review only - http://bmjopen.bmj.com/site/about/guidelines.xhtml

overt realisation of the impact of the ‘remoteness’ on the likelihood of getting a timely ambulance response:

“If it was very severe, I would get my husband… to drive me to the hospital”. [Ahl et al., participant quotation]

“Patients in rural areas consult primary health care less frequently than urban patients… and are more likely to call their own general practitioner in emergencies” [Ahl et al., author interpretation]

Themes in the practical domain also allude to an undertone of frustration that patients can experience if they do not initially receive the care that they expect. Being unable to ‘get through’ to the right person first time seems to link with the negative emotional responses associated with inability to get reassurance, worsening uncertainty and anxiety and a feeling that the ‘system doesn’t care’. Free codes relating to practical barriers and access issues frequently co-existed with codes describing negative emotions:

“The walk in centre just sends you up to hospital if they can’t do anything. So you end up, like, waiting 3 hours twice!” [Booker et al., participant quotation]

“The district nurse said she couldn’t help. The doctor’s surgery couldn’t help. The hospital clinic couldn’t help. After that many calls, you just snap?” [Booker et al, participant quotation].

Within the emotional domain there were also descriptive themes and codes relating to the *potential* treatment that the ambulance service could provide, if necessary. Strong links existed between patients’ knowledge of practical treatment that crews could provide, and the emotional themes of reassurance and uncertainty. This suggests that it is as much the *potential* and *perceived* capacity of the ambulance

For peer review only - http://bmjopen.bmj.com/site/about/guidelines.xhtml

service to deliver life-saving treatment that is valued, and alludes to the idea of risk-avoidance or risk-minimisation when selecting which urgent care service to access.

The Emotional Domain

This domain encompassed descriptive themes relating to how people responded emotionally to the experience of needing or receiving ambulance treatment. Descriptive themes in this domain were organised into three groups: the issue of uncertainty about their condition or health, the need for (and impact of) reassurance, and the effect of compassion shown by others during the ambulance service contact. Analysis of emotion-related themes across the studies supports the ideas that the emotional response was not always congruent with actual medical need.

The descriptive theme of uncertainly was often associated with codes referencing anxiety, but appears to more broadly reflect the idea of dealing with the unknown:

"I just needed someone there quickly… to tell me whether it was something serious?".
[Booker et al., participant quotation]

"A sense of insecurity and vulnerability emerged due to now knowing what was wrong"
[Rantala et al., author interpretation]

Within the descriptive theme of uncertainty there existed a continuum of emotions, ranging from codes associated with minor indecision through to despair. There was no clear or consistent link with the actual type or severity of clinical condition experienced by these patients, suggesting that extreme emotional responses were apparent in even relatively minor medical problems. Importantly, uncertainty appears

For peer review only - http://bmjopen.bmj.com/site/about/guidelines.xhtml

to be experienced by ambulance crews as well, particularly around how best to manage non-urgent presentations:

"Even where the crew did not think that the patient should go to hospital and the patient did not want to go, crew members were still bothered by the fear of a possible comeback if it turned out to be the wrong decision". [Porter et al., author interpretation]

This is particularly noteworthy, as the descriptive theme of reassurance links equally with themes that rest principally in the practical domain and themes that appear purely emotion-driven. This suggests the potential powerful effect of reassurance – perhaps almost therapeutic - on helping patients gain practical *and* emotional control of the situation they are experiencing:

"I felt so ill that I thought I was about to become unconscious, but knowing that someone is on the way meant I was not worried, I knew they would find me". [Ahl et al., participant quotation]

"The stress and pain began to ease when I knew that the ambulance was on its way" [Ahl et al., participant quotation]

Also closely linked with reassurance and uncertainty is the descriptive theme of compassion. Codes within the compassion theme sometimes occurred alongside codes within the practical 'ambulance as transport' theme, but in a manner that suggests at least some patients do see these two roles as distinct:

"Well, they took care of me and put me on a stretcher, they checked the whole time that I was all right so it sure was more care than transport". [Ahl et al., participant quotation]

For peer review only - http://bmjopen.bmj.com/site/about/guidelines.xhtml

For peer review only

The perceived absence of compassion when calling for an ambulance was also noteworthy, impacting upon the overall experience:

“He didn’t care. It was just when I was speaking to him he wasn’t sort of listening”. [Togher et al., participant quotation]

Such codes occurred commonly with examples of clear mismatch between the ambulance clinician’s assessment and the patient’s assessment of medical need. In such circumstances the emotional function of the ambulance service was not fulfilled, even if the medical function was.

The Process Domain

Central to this analysis is the third analytical theme of the ‘processes’ involved in balancing the practical domain with the emotional domain. This analytical theme relates the emotional aspects of seeking urgent help with the practicalities of obtaining and receiving ambulance treatment, by describing processes central to help-seeking and decision-making in this context. These ‘process’ descriptive themes were grouped into three: being influenced in the decision-making by others, being empowered to take responsibility for one’s health, and taking or handing over control of the situation. This analysis demonstrated the frequent juxtaposition – even tension – of some of the themes related to practicalities with themes related to emotional responses.

A strong descriptive theme running through the analysis of the process domain was the idea of patients achieving some sense of control over a perceived urgent or distressing situation by phoning an ambulance:

For peer review only - http://bmjopen.bmj.com/site/about/guidelines.xhtml

For peer review only

"When patients phone for an ambulance, they hope to gain control of the experienced emergency situation based on trust and the expectation that they will be assisted… which implies an ethical demand." [Rantala et al., author interpretation]

"It feels good once you have made the decision and phoned for an ambulance. They know that something is wrong… now I don't have to take further responsibility" [Ahl et al., participant quotation]

The approach of decisively 'taking control' of the situation appears to be used by some patients to bring about a definitive resolution to an otherwise intolerable situation, by accessing ambulance treatment. Contrastingly, some patients appear much more comfortable with handing over control of a situation they don't fully understand to the ambulance service, thereby exonerating themselves of responsibility for managing it. This concept of 'responsibility' appears quite complex. Linked with the emotional theme of reassurance is a need to also be 'reassured' that the decision to call an ambulance was correct – to seek legitimisation for using a finite (even scarce) resource for one's own needs. When callers 'hand over' this practical element of the decision-making by calling the emergency number, they appear to feel emotionally more comfortable with the outcome of an ambulance attending, as it is not directly they who made the decision to send one. Rather it is the *system* that has decided that an ambulance is necessary, and is responsible for the outcome – there appears a degree of conceptual disconnect between calling for an ambulance and receiving one.

It is also clear how powerful the influence of others (particularly family) can also be on this particular aspect of effectively 'handing over' the decision-making process:

For peer review only - http://bmjopen.bmj.com/site/about/guidelines.xhtml

“It was my wife who was very concerned and wanted to make the call”. [Rantala et al., participant quotation]

A particularly important aspect of this descriptive theme is that the ‘influencer’ doesn’t even necessarily have to be present at the time of the need for treatment for their views to form part of the decision-making:

“I could just hear my daughter saying “oh mum, why didn’t you get the ambulance out?” even though I didn’t think I needed it.” [Booker et al., participant quotation]

Ambulance crews also have an ability to exert influence on the patients’ decision-making, particularly around whether hospitalisation is necessary or not. Particularly noteworthy in the analysis is that the process of exerting this influence – whilst often conveyed in very practical terms (such as the likely lengthy waiting around in hospital for a bed to be available) – has the ability to produce a strong emotional response. This emotional response can often be one of empowerment and taking responsibility, through mutual respect, partnership and shared decision-making:

“[Paramedics] also talked a great deal about the possibility of persuading people to stay at home when hospital treatment was not necessary” [Porter et al., author interpretation]

“So I was respected as a full partner in the conversation. It was a very nice experience” [Rantala et al., participant quotation]

But the process of influencing can also result in negative emotional responses such as guilt, which may even have direct consequences on the patient’s choice of care in the future. Commonly the codes associated with negative influences occurred in encounters that were being labelled as ‘inappropriate’:

For peer review only

For peer review only - http://bmjopen.bmj.com/site/about/guidelines.xhtml

“Afterwards I felt very guilty about what happened and why I had done it. I just hope I don’t have to call them again.” [Rantala et al., participant quotation]

“One feels so worthless… they just don’t believe me!” [Rantala et al., participant quotation]

For peer review only

For peer review only - http://bmjopen.bmj.com/site/about/guidelines.xhtml

DISCUSSION

This qualitative synthesis suggests that the decision-making processes involved in calling for and receiving ambulance treatment for a ‘primary care sensitive’ problem are shaped by a combination of emotional and practical factors. Often, the ambulance service contact functions to fulfil *both* emotional and practical needs in the caller. Regardless of the actual severity of the precipitating clinical condition, both of these groups of factors play an important role in choosing (and valuing) ambulance-delivered care. This synthesis proposes that three key processes are involved in combining emotional and practical factors: handing over of control and responsibility, the influencing by important others, and empowerment to take responsibility for one’s own health. Informal carers, relatives and health professionals can all be ‘influencers’, and do not even necessarily have to be physically present for their ‘influence’ to be considered by a patient.

There is currently discussion in the research and policy literature about how to define and reduce so-termed ‘unnecessary’ or ‘inappropriate’ ambulance use. This synthesis supports previous findings [5] that suggest the concept of ‘inappropriate’ ambulance use is far more nuanced than a definition based purely upon the clinical condition that a patient receives treatment for. This analysis suggests there is a strong association with negative emotional responses when callers believe they are being labelled as ‘inappropriate’. The implications of these responses are complex, as there is a clear need to challenge inefficient (or irresponsible) use of the finite emergency health resources that are under increasing pressure, and to promote personal health resilience strategies. However, this must be balanced with an understanding of what prompted the contact, so that additional barriers are not constructed that may adversely impact future help-seeking for serious emergency situations. The themes from this review suggest that urgent care triage systems need

For peer review only - http://bmjopen.bmj.com/site/about/guidelines.xhtml
For peer review only

to be sensitive to both the perceived emotional and practical drivers of a call for help, but importantly need to recognise how this may be shaped by carers and relatives. Whilst the ambulance service contact is sometimes therapeutic in itself, this synthesis highlights the on-going conflict that ambulance services face with directing finite resources to resolving an individual's (potentially emotionally driven) need, verses the wider emergency care needs of the population. This synthesis suggests that the components of the treatment contact that patients value are those that support their own ability to 'process' both the emotional and practical challenges they are presented with by their health condition, and enable them to find a comfortable level of control of the situation and possible outcomes. It is possible that these valued components might be deliverable in ways other than despatching emergency ambulances to physically attend.

Conclusions

Supporting patients and their relatives to access the "right advice in the right place, first time" has underpinned UK national urgent care policy for nearly a decade [26], but is hampered by an incomplete understanding of how patients conceptualise urgency. This synthesis builds on recent work in the emergency department setting, that suggested patients define situations worthy of emergency health resources according to socio-emotional factors rather than purely the symptoms or physiology underlying their illness.[27] This synthesis also suggest that the 'organisation incentives' and 'secondary benefits' of the health system referred to in the classic sociological models of illness and help-seeking [8] may also exist when choosing ambulance care via the processes of legitimisation and, perhaps somewhat paradoxically, empowerment. Previous work has acknowledged how practical issues such as rurality and remoteness,(e.g.[28,29]) perceptions of out of hours primary care,(e.g.[30,31]) and views of telephone advice services [32] shape decision-

For peer review only - http://bmjopen.bmj.com/site/about/guidelines.xhtml

making. This synthesis highlights how importantly patients regard the views of those around them, and how emotionally conflicted some patients can be about their care choices.

Practice Implications

This qualitative systematic review is the result of a comprehensive, systematic literature search in accordance with a prospectively published protocol. Although the resulting synthesis is based on a relatively small number of studies meeting the inclusion criteria, this reflects a relatively under-researched area of interest. The majority of included studies are from UK-based ambulance services, and all were undertaken in a European system with a primary health care model. As such, there may be some limitations in applicability to contrasting ambulance systems. Additionally, all reported studies were based on adult populations, limiting the conclusions that can be drawn for paediatric populations. Some of the included studies, whilst including relevant data about ambulance use for 'primary care sensitive' conditions, were not designed specifically to explore these groups. Despite these limitations, this study is, to the authors' knowledge, the first synthesis of qualitative evidence combining patients', carers' and health professionals' perspectives on the use of ambulances for lower-acuity conditions. Future work needs to explore how urgent care systems can be sensitive and responsive to the emotional component of the help-seeking process, whilst still providing the practical assistance that patients and carers require. This necessarily will involve a balanced approach that empowers urgent healthcare providers to tailor the format of the response accordingly. Additionally, with increasing pressures on ambulance services, there is a need for specific research exploring how to empower patients and carers towards resilient, resource-responsible help-seeking strategies in these situations.

For peer review only - http://bmjopen.bmj.com/site/about/guidelines.xhtml

For peer review only

References

[1] Pickering A, Mason S, Turner J, et al. A comparative review of ambulance service best practice. Office of the Strategic Health Authorities. http://www.nhsimas.nhs.uk/fileadmin/Files/IST/ESR_A_comparative_review_of_intrnational_ambulance_service_best_practice.pdf Access Date: March 2017

[2] Department of Health, Taking healthcare to the patient: transforming NHS Ambulance Services. http://ircp.info/Portals/11/Future/NHS%20EMS%20Policy%Recommendation2005.pdf Access Date: March 2017

[3] Department of Health, Tackling demand together. A toolkit for improving urgent and emergency care pathways by understanding increases in 999 demand. http://www.dh.gov.uk/prod_consum_dh/groups/dh_digitalassets/documents/digitalasset/dh_106924.pdf Access Date: March 2017

[4] NHS England, High quality care for all, now and for future generations: transforming urgent and emergency care services in England. The evidence base from the urgent and emergency care review. http://www.england.nhs.uk/wp-content/uploads/2013/review Access Date: March 2017

[5] Booker MJ, Shaw ARG, Purdy S. Why do patients with 'primary care sensitive' problems access ambulance services? A systematic mapping review of the literature. *BMJ Open* 2015;5:e007726.

For peer review only - http://bmjopen.bmj.com/site/about/guidelines.xhtml

[6] Durand AC, Palazzolo S, Tanti-Hardouin N, et al. Nonurgent patients in emergency departments: rational or irresponsible consumers? Perceptions of professionals and patients. *BMC Research Notes* 2012;5:525.

[7] Booker MJ, Simmonds RL, Purdy S. Patients who call ambulance for primary care problems: a qualitative study of the decision-making process. *Emerg Med J* 2014;31:448-52.

[8] Mechanic D. Sociological Dimensions of Illness Behavior. *Soc Sci Med* 1995;41:1207–16.

[9] Hunter C, Chew-Graham C, Langer S, et al. A qualitative study of patient choices in using emergency health care for long-term conditions: the importance of candidacy and recursivity. *Patient Educ Couns* 2013;93:335-41.

[10] Langer S, Chew-Graham C, Hunter C, et al. Why do patients with long-term conditions use unscheduled care? A qualitative literature review. *Health and Social Care in the Community* 2013;21:339-51.

[11] Thomas J, Harden A. Methods for the thematic synthesis of qualitative research in systematic reviews. *BMC Medical Research Methodology* 2008;8:45.

[12] Strauss AL, Corbin J. Basics of qualitative research: grounded theory procedures and techniques. Newbury Park, CA: Sage 1990

[13] Tong A, Flemming K, McInnes E, et al. Enhancing transparency in reporting the synthesis of qualitative research: ENTREQ. *BMC Med Res Methodol* 2012;12:181.

For peer review only - http://bmjopen.bmj.com/site/about/guidelines.xhtml

For peer review only

[14] Campbell R, Pound R, Pope C, et al. Evaluating meta-ethnography: a synthesis of qualitative research on lay experiences of diabetes and diabetes care. *Soc Sci Med* 2003;56:671-84.

[15] Dixon-Woods M, Cavers D, Agarwal S, et al. Conducting a critical interpretive synthesis of the literature on access to healthcare by vulnerable groups. *BMC Med Res Methodol* 2006;6:35-47.

[16] Seale C. Quality in qualitative research. *Qual Inq* 1999;5:465-78.

[17] Spencer L, Richie J, Lewis J, et al. Quality in qualitative evaluation: a framework for assessing research evidence. London: Cabinet Office 2003.

[18] Walsh D, Downe S. Appraising the quality of qualitative research. *Midwifery* 2006;22:108-19.

[19] Critical Appraisal Skills Programme (CASP) CASP Checklists. Oxford: UK. http://www.casp-uk.net/#!casp-tools-checklists/c18f8 Access Date March 2017

[20] Downe S. Metasynthesis: a guide to knitting smoke. *Evidence Based Midwifery* 2008;6:4-8.

[21] Ahl C, Nystrom M, Jansson L. Making up one's mind: patients' experiences of calling an ambulance. *Accid Emerg Nurs* 2006;14:11-19.

[22] Campbel NC, Iversen L, Fermer J. A qualitative study in rural and urban areas on whether – and how – to consult during routine and out of hours. *BMC Family Practice* 2006;7:26.

For peer review only

For peer review only - http://bmjopen.bmj.com/site/about/guidelines.xhtml

[23] Rantala A, Ekwall A, Forsberg A. The meaning of being triaged to non-emergency ambulance care as experienced by patients. *Int Emerg Nurs* 2016;25:65-70.

[24] Porter A, Snooks H, Youren A, et al. 'Should I stay or should I go?' Deciding whether to go to hospital after a 999 call. *J Health Serv Res Policy*. 2007;S1:32-8.

[25] Togher FJ, O'Cathain A, Phung VH, et al. Reassurance as a key outcome valued by emergency ambulance service users: a qualitative interview study. *Health Expectations* 2015;18:2951-61.

[26] Department of Health. NHS next stage review: Our vision for primary and community care. http://www.nhshistory.net/dhvisionphc.pdf Access Date March 2017

[27] Morgans A, Burges SJ. What is a health emergency? The difference in definition and understanding between patients and health professionals. *Australian Health Review* 2011;35:284-9.

[28] Farmer J, Iversen L, Campbell NC, et al. Rural/urban differences in accounts of patients' initial decisions to consult primary care. *Health & Place* 2006;12:210-21.

[29] Turnbull J, Pope C, Martin D, et al. Do telephones overcome geographical barriers to general practice out-of-hours services? Mixed-methods study of parents with young children. *Journal of Health Services Research and Policy* 2010;15:21-7.

For peer review only - http://bmjopen.bmj.com/site/about/guidelines.xhtml

[30] Egbunike JN, Shaw C, Bale S, et al. Understanding patient experience of out-of-hours general practitioner services in South Wales: a qualitative study. *Emerg Med J* 2008;25:649-54.

[31] Foster J, Dale J, Jessopp L. A qualitative study of older people's views of out-of-hours services. *BJGP* 2001;51:719-23.

[32] O'Cathain A, Knowles E, Turner J, et al. Acceptability of NHS 111 the telephone service for urgent health care: cross sectional postal survey of users' views. *Family Practice* 2014;31:193-200.

For peer review only

CONTRIBUTORS

MJB is the main author involved in all stages of the design, analysis and write-up. ARGS and SP contributed to the methodological approach, data extraction, verification and analysis through regular research meetings. ARGS and SP contributed significant input to the written paper. All authors have approved the final version of the paper ahead of publication.

ACKNOWLEDGMENTS

None.

COMPETING INTERESTS

None declared.

FUNDING

MJB is funded by a National Institute for Health Research Doctoral Research Fellowship. This paper presents independent research funded by the National Institute for Health Research (NIHR). The views expressed are those of the author(s) and not necessarily those of the NIHR, the NHS or the Department of Health.

DATA SHARING STATEMENT

No additional data are available.

For peer review only

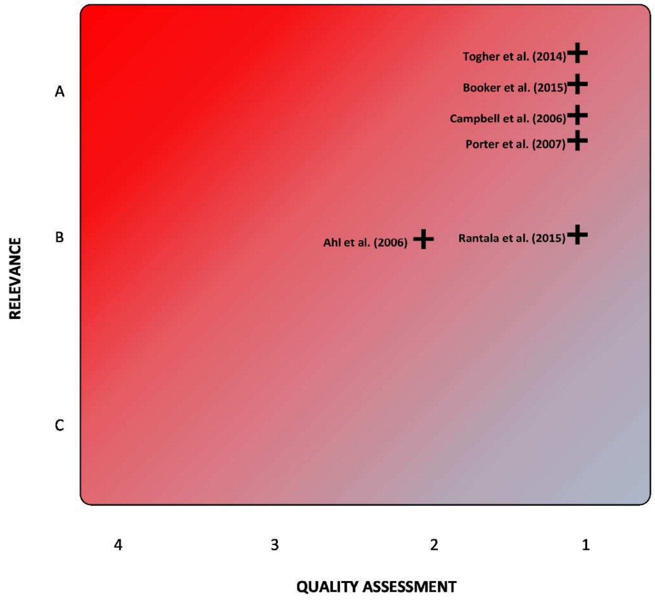

'Quality-Relevance plot' of included studies.

209x297mm (300 x 300 DPI)

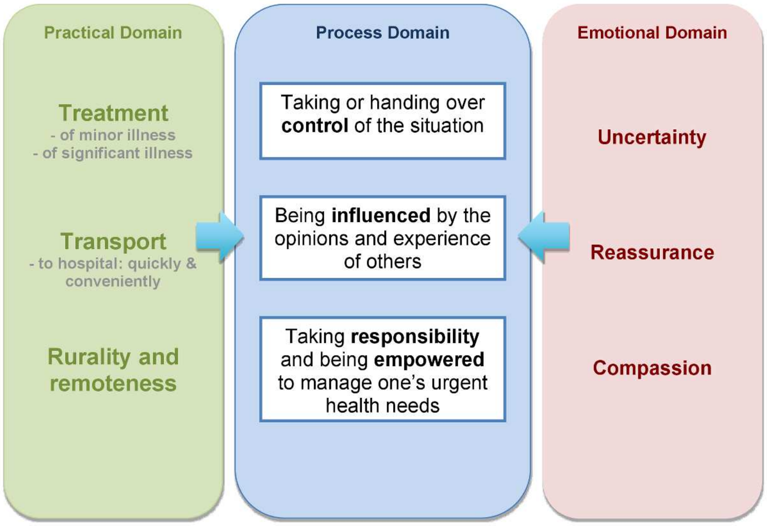

Relationships between analytical themes

209x297mm (300 x 300 DPI)

Appendix – Search String Example, PubMed

byDateRange: 01/01/1980-30/06/2014;
((((((((((((((((emergency medical services
[mesh: EXP Emergency Health Services; Emergency Services, Medical; Emergicentres; Medical Services, Emergency; Prehospital Emergency Care; Services, Emergency Medical])
OR emergency care* [tiab])
OR prehospital* [tiab])
OR pre#hospital* [tiab])
OR pre*hospital* [tiab])
OR EMS [tiab]) OR emerg* serv* [tiab])
OR ambulances
[mesh: EXP Emergency Mobile Units; Mobile Emergency Units])
OR ambulance* [tiab])
OR 999 [tiab])
OR *111 [tiab])
OR 911 [tiab])
OR paramedic* [tiab])
OR emerg* med* tech* [tiab])
OR EMT [tiab])
OR emergency treatment
[mesh: EXP Emergency Therapy; Therapy, Emergency]) OR emergency* [tiab])
OR emergencies [mesh])
AND
((((((((primary health care
[mesh: EXP Primary Care; Primary Healthcare])
OR primary care* [tiab])
OR primary *care [tiab])
OR family medicine* [tiab])
OR family practice* [tiab])
OR after hours care
[mesh: EXP After Hours Care; Out Of Hours Medical Care])
OR OOH [tiab])
OR out of hours* [tiab])
OR out#of#hours [tiab])
OR primary care sensitive [tiab])

For peer review only

OR primary care problem [tiab])

OR general practice

[mesh: EXP General Practitioners]))

OR (((((((social care* [tiab])

OR non*serious* [tiab])

OR non*emergenc* [tiab])

OR non*urgent [tiab])

OR minor* [tiab])

OR routine* [tiab])

OR avoid* [tiab])

OR inappropriate* [tiab])

OR unnecessary* [tiab])

{1381}

For peer review only

PRISMA 2009 Checklist

Section/topic	#	Checklist item	Reported on page #
**TITLE**			
Title	1	Identify the report as a systematic review, meta-analysis, or both.	1
**ABSTRACT**			
Structured summary	2	Provide a structured summary including, as applicable: background; objectives; data sources; study eligibility criteria, participants, and interventions; study appraisal and synthesis methods; results; limitations; conclusions and implications of key findings; systematic review registration number.	2, 6-9
**INTRODUCTION**			
Rationale	3	Describe the rationale for the review in the context of what is already known.	4-5
Objectives	4	Provide an explicit statement of questions being addressed with reference to participants, interventions, comparisons, outcomes, and study design (PICOS).	6
**METHODS**			
Protocol and registration	5	Indicate if a review protocol exists, if and where it can be accessed (e.g., Web address), and, if available, provide registration information including registration number.	6
Eligibility criteria	6	Specify study characteristics (e.g., PICOS, length of follow-up) and report characteristics (e.g., years considered, language, publication status) used as criteria for eligibility, giving rationale.	6-7
Information sources	7	Describe all information sources (e.g., databases with dates of coverage, contact with study authors to identify additional studies) in the search and date last searched.	6
Search	8	Present full electronic search strategy for at least one database, including any limits used, such that it could be repeated.	6, Appendix 1
Study selection	9	State the process for selecting studies (i.e., screening, eligibility, included in systematic review, and, if applicable, included in the meta-analysis).	6-7
Data collection process	10	Describe method of data extraction from reports (e.g., piloted forms, independently, in duplicate) and any processes for obtaining and confirming data from investigators.	7-8
Data items	11	List and define all variables for which data were sought (e.g., PICOS, funding sources) and any assumptions and simplifications made.	7-8
Risk of bias in individual studies	12	Describe methods used for assessing risk of bias of individual studies (including specification of whether this was done at the study or outcome level), and how this information is to be used in any data synthesis.	9-11
Summary measures	13	State the principal summary measures (e.g., risk ratio, difference in means).	N/A

For peer review only

PRISMA 2009 Checklist

Section/topic	#	Checklist item	Reported on page #
Synthesis of results	14	Describe the methods of handling data and combining results of studies, if done, including measures of consistency (e.g., I^2) for each meta-analysis.	7,13

Section/topic	#	Checklist item	Reported on page #
Risk of bias across studies	15	Specify any assessment of risk of bias that may affect the cumulative evidence (e.g., publication bias, selective reporting within studies).	9-11
Additional analyses	16	Describe methods of additional analyses (e.g., sensitivity or subgroup analyses, meta-regression), if done, indicating which were pre-specified.	N/A
**RESULTS**			
Study selection	17	Give numbers of studies screened, assessed for eligibility, and included in the review, with reasons for exclusions at each stage, ideally with a flow diagram.	12
Study characteristics	18	For each study, present characteristics for which data were extracted (e.g., study size, PICOS, follow-up period) and provide the citations.	12
Risk of bias within studies	19	Present data on risk of bias of each study and, if available, any outcome level assessment (see item 12).	N/A
Results of individual studies	20	For all outcomes considered (benefits or harms), present, for each study: (a) simple summary data for each intervention group (b) effect estimates and confidence intervals, ideally with a forest plot.	N/A
Synthesis of results	21	Present results of each meta-analysis done, including confidence intervals and measures of consistency.	N/A
Risk of bias across studies	22	Present results of any assessment of risk of bias across studies (see Item 15).	N/A
Additional analysis	23	Give results of additional analyses, if done (e.g., sensitivity or subgroup analyses, meta-regression [see Item 16]).	N/A
**DISCUSSION**			
Summary of evidence	24	Summarize the main findings including the strength of evidence for each main outcome; consider their relevance to key groups (e.g., healthcare providers, users, and policy makers).	25-27
Limitations	25	Discuss limitations at study and outcome level (e.g., risk of bias), and at review-level (e.g., incomplete retrieval of identified research, reporting bias).	3, 27
Conclusions	26	Provide a general interpretation of the results in the context of other evidence, and implications for future research.	26-27
**FUNDING**			
Funding	27	Describe sources of funding for the systematic review and other support (e.g., supply of data); role of funders for the systematic review.	33

From: Moher D, Liberati A, Tetzlaff J, Altman DG, The PRISMA Group (2009). Preferred Reporting Items for Systematic Reviews and Meta-Analyses: The PRISMA Statement. PLoS Med 6(7): e1000097. doi:10.1371/journal.pmed1000097

For peer review only - http://bmjopen.bmj.com/site/about/guidelines.xhtml

PRISMA 2009 Checklist

For more information, visit: www.prisma-statement.org.

For peer review only
